# Roles of Major Facilitator Superfamily Transporters in Phosphate Response in *Drosophila*

**DOI:** 10.1371/journal.pone.0031730

**Published:** 2012-02-16

**Authors:** Clemens Bergwitz, Matthew D. Rasmussen, Charles DeRobertis, Mark J. Wee, Sumi Sinha, Hway H. Chen, Joanne Huang, Norbert Perrimon

**Affiliations:** 1 Endocrine Unit, Massachusetts General Hospital, Boston, Massachusetts, United States of America; 2 Department of Genetics, Harvard Medical School/Howard Hughes Medical Institute, Boston, Massachusetts, United States of America; 3 Computer Science and Artificial Intelligence Lab, Massachusetts Institute of Technology, Boston, Massachusetts, United States of America; National Cancer Institute, United States of America

## Abstract

The major facilitator superfamily (MFS) transporter *Pho84* and the type III transporter *Pho89* are responsible for metabolic effects of inorganic phosphate in yeast. While the *Pho89* ortholog *Pit1* was also shown to be involved in phosphate-activated MAPK in mammalian cells, it is currently unknown, whether orthologs of *Pho84* have a role in phosphate-sensing in metazoan species. We show here that the activation of MAPK by phosphate observed in mammals is conserved in *Drosophila* cells, and used this assay to characterize the roles of putative phosphate transporters. Surprisingly, while we found that RNAi-mediated knockdown of the fly *Pho89* ortholog *dPit* had little effect on the activation of MAPK in *Drosophila* S2R+ cells by phosphate, two *Pho84*/SLC17A1–9 MFS orthologs (MFS10 and MFS13) specifically inhibited this response. Further, using a *Xenopus* oocyte assay, we show that MSF13 mediates uptake of [^33^P]-orthophosphate in a sodium-dependent fashion. Consistent with a role in phosphate physiology, MSF13 is expressed highest in the *Drosophila* crop, midgut, Malpighian tubule, and hindgut. Altogether, our findings provide the first evidence that *Pho84* orthologs mediate cellular effects of phosphate in metazoan cells. Finally, while phosphate is essential for *Drosophila* larval development, loss of MFS13 activity is compatible with viability indicating redundancy at the levels of the transporters.

## Introduction

Inorganic phosphate, the mono- or divalent anion of phosphoric acid [HPO_4_^3−^, H_2_PO_4_^2−^], is required for cellular functions such as DNA and membrane lipid synthesis, generation of high-energy phosphate esters, and intracellular signaling [1]. Disturbances of phosphate homeostasis are serious human disorders [2]: the clinical consequences of severe hypophosphatemia, which for example is seen in severe malnutrition or tumor-induced hypophosphatemia [3], include hemolysis, skeletal muscle myopathy, cardiomyopathy, neuropathy, osteomalacia and, in some cases contribute to death. Hyperphosphatemia on the other hand leads to tissue calcifications and metabolic changes, which are to date poorly understood. Hyperphosphatemia is encountered most frequently in patients with chronic kidney disease (CKD), which affects 20 Million Americans today, and the serum phosphate level is an important predictor of mortality in this population [4], [5], [6]. It is also seen in familial hyperphosphatemic tumoral calcinosis, a human disorder that was recently attributed to loss-of function mutations in the genes encoding fibroblast growth factor 23 (*FGF23*), UDP-GalNAc transferase 3 (*GALNT3*), and Klotho (*KL*) [7]. Furthermore, mouse models with hyperphosphatemia due to loss-of-function mutations in *Fgf23*, *Kl* or *Galnt3* die prematurely unless they are placed on a phosphate-restricted diet to improve their lifespan [8], [9], [10] and it is possible that similar mechanisms underlie the known beneficial effects of dietary phosphate-restriction in humans with CKD. An understanding of the molecular basis underlying the metabolic and endocrine phosphate effects is therefore of great significance for human disease.

The intracellular concentration of inorganic phosphate is maintained by membrane transporters which accumulate phosphate against an electrochemical gradient coupled to the plasma membrane H^+^ [11] or Na^+^ gradients [12], at concentrations larger than would be predicted if phosphate were distributed passively across the membrane. Much has been learned about phosphate transport in bacteria and in yeast. Bacteria sense phosphate using a four-component Pst-transporter (*PstS, PstA, PstB, PstC*), which is similar to mammalian ABC transporters. Binding of phosphate to *PstSABC* represses a two component signaling system composed of the sensory histidine kinase *PhoR* and the winged helix transcription factor *PhoB* [13], [14]. Different from bacteria, the main phosphate-sensing transporter *Pho84* in yeast belongs to the major facilitator family (MFS) which regulates the cyclin/cyclin-dependent kinase (CDK) complex *Pho80–Pho85* [15]. The activity of *Pho80–Pho85* in turn regulates the subcellular localization of the basic helix-loop-helix transcription factor *Pho4,* which belongs to the myc family. Interestingly, a number of transporters, *Pho87, 89, 90* and *91*, can compensate for loss of *Pho84* under certain conditions in yeast suggesting that signaling is independent of the mode of cellular uptake, and that intracellular phosphate is the signal for gene-regulation [16]. However, the fact that overexpression of a phosphate-transport deficient *Pho84* variant can rescue regulation of the extracellular alkaline phosphatase *Pho5* by phosphate in *Pho84* deficient strains, while overexpression of *Pho87*, *Pho90*, *Pho91* or *Pit* is ineffective, suggests, that binding of extracellular phosphate alone may be sufficient for some downstream effects of phosphate [16].

Humans have three types of membrane-bound phosphate transporters: The type I transporters *SLC17A1–9* that belong to the MFS group. MFS are widely expressed and some also mediate transport of organic anions, such as uric or sialic acid, or certain antibiotics [17], [18]. Conversely, the human type II phosphate transporters *NPT2a*, *NPT2b*, and *NPT2c*, and type III phosphate transporters *Pit1* and *Pit2* are thought to be exclusively transporting phosphate [12], [19]. *NPT2a*, *NPT2c*, and *Pit2* are expressed in the renal proximal tubule and mediate re-absorption of phosphate from the urine, *NPT2b* and *Pit2* mediate absorption of phosphate from the diet in the gut, and Pit1 is ubiquitously expressed and facilitates uptake of phosphate from the circulation to supply cellular functions [20], [21]. *Pho84* belongs to the MFS group, *Pho87, 90* and *91* are related to metazoan sodium-sulfate transporters (*SLC13A1–4*), and *Pho89* is related to the type III sodium-phosphate transporters *SLC20A1* (*Pit*-1) and *SLC20A2* (*Pit*-2) [22], [23].

Compared to bacteria and yeast, little is known about the metabolic effects of phosphate in metazoan species [20], [21]. Over the past decade, activation of MAPK by inorganic phosphate at concentrations between 5–10 mM alone was demonstrated in multiple cell lines including MC3T3 mouse fibroblast cells [24], [25], chondrogenic ATDC5 cells, MC3T3-E1 osteoblasts and ST2 murine bone marrow stromal cells [26], HEK293 human proximal tubular cells [27], and lung alveolar cells [28]. Although some cell lines, for example C2C12 or L929 cells, are less responsive than others [26], activation of MAPK by phosphate appears to be quite universal. Addition of phosphonoformic acid (PFA), a competitive antagonist of phosphate transporters and cellular phosphate uptake [29], [30], or siRNA-mediated knockdown of the type III transporter *Pit1* blocks activation of MAPK by phosphate in HEK293 cells [27]. Furthermore, using cell lines expressing a Pi-transport-deficient *Pit1* transporter, Beck et al. recently reported that *Pit1* may have transport-independent effects on cell proliferation and tumor growth *in vitro* and *in vivo*, although it remains to be shown whether these effects depend on phosphate-binding to *Pit1* [31]. Targeted deletion, hypomorphic and overexpression mutants of *Pit1* support a role of this transporter in liver growth and phosphate homeostasis [32], [33], [34], however, surprisingly, *Pit1* null mice showed normal embryonic and fetal development. Collectively, these data suggest an important role of the type III transporter *Pit1* in mammalian phosphate-sensing, but it remains unclear whether phosphate is required to enter the cell to activate an intracellular sensor, whether it binds extracellularly, or whether multiple transporters are involved.

In multicellular organisms the circulating phosphate levels and total body phosphate content are tightly regulated by a number of hormones, including parathyroid hormone (PTH), 1,25-dihydroxy vitamin D (1,25(OH)_2_D), and fibroblast growth factor 23 (FGF23). Serum phosphate feeds back to regulate these factors in an endocrine fashion [2] with high phosphate increasing the secretion of PTH and FGF23 and low phosphate stimulating the synthesis of 1,25(OH)_2_D, the active form of vitamin D [21]. Owing to the lack of suitable cell lines to permit the study of the synthesis and secretion of these hormones *in vitro*, it is currently unknown whether the MAPK pathway is involved in these endocrine effects of phosphate. Thus, it remains unclear whether the “metabolic” and the “endocrine” effects of phosphate use the same or different signal transduction cascades.

To understand whether phosphate-induced MAPK is evolutionarily conserved, we investigated the response to phosphate in *Drosophila* cells. We show that, as in mammalian cells, phosphate activates MAPK in fly cells, and used this assay to identify members of the MFS of transporters involved in sensing phosphate. Our findings indicate that two type I sodium-phosphate *Pho84*/SLC17A1–9 MFS orthologs (MFS10 and MFS13) mediate some of the cellular effects of phosphate, a finding which may be relevant to higher species and humans.

## Materials and Methods

### Cell culture

The *Drosophila* hemocyte cell lines S2R+, a variant adherent S2 line [35], and Kc167 cells [36] were cultured in Schneider's *Drosophila* medium (Invitrogen) supplemented with 10% heat-inactivated fetal bovine serum (Hyclone, Fisher Scientific) and penicillin/streptomycin at 25 C using a humidified incubator.

Routine culture was performed in Schneider's medium containing 7 mM sodium-phosphate buffer (pH 7.4). Cells were sub-cultured with a cell scraper and plated at 200.000 cells/well in 48-well plates. After 48-hr. culture in phosphate-free Schneider's medium (Invitrogen) containing 10% FBS (final phosphate concentration about 100 uM) in 48-multiwell plates, cells were pretreated with phosphonoformic acid (PFA) 5–30 mM (Sigma), Ly294002 50 uM (Sigma), or UO126 30 uM (Sigma) for 60 min., followed by stimulation with sodium phosphate buffer 1–10 mM (pH7.4), 10 mM Na-sulfate (pH 7.4), or human insulin 25 ug/ml (Sigma) for 1–30 min. The phosphate concentration in phosphate-free medium was sufficient to permit survival of S2R+ cells, although proliferation rate was somewhat slower, as indicated by Trypan blue staining, and their responses to insulin and phosphate.

### Western blot analysis

Following pretreatment and stimulation the 48-well plate was placed on ice and the culture medium was aspirated. Cells were then lysed with 50 ul lysis buffer (62.5 mM Tris HCL (pH 6.8), 1% SDS, 1 mM EDTA, 1 mM EGTA, 0.05 TiU/ml aprotonin, 1 M PMSF, 100 mM Na-orthovanadate, 0.8% SDS, 3.2% glycerol, 2% beta mercaptoethanol, 0.0015% bromephenolblue).

15 ul cell lysates were then separated on 12% Tris-HCl SDS-polyacrylamide, electro-transferred to PVDF membranes and hybridized with anti-phospho-ERK1/2 #9106 or #4730 or total-ERK1/2 antibody #4695 (Cell signaling) in phosphate buffered saline containing 0.1% Tween 20 (PBST) and 5% non-fat dry milk at 4 C over night. On the following day, a developing reaction with horseradish-peroxidase conjugated secondary antibodies in PBST+5% non-fat dry milk was preformed at room temperature for 60 min. to permit detection of the phospho- or total-ERK signal using chemiluminscence/autoradiography (Perkin Elmer). Following densitometric analysis of the autoradiograms, phospho/total ERK ratios were converted into fold over basal to permit statistical evaluation of pooled Western blot experiments.

### Phylogenetic analysis

We downloaded protein sequences from Ensembl version 56 [37] and used BLAST to cluster all known yeast, *Drosophila* and human proteins containing the MFS protein domain PF07690 [38] into five main families. Next using Bayesian phylogenetic reconstruction (MrBayes v3.1.2 [39]) we identified 29 fly orthologs that are most closely related to yeast Pho84 and human SLC17A1–9, an anion transporter subfamily with members known to mediate phosphate transport. Refer to Table S2 for all blast hits S. cerevisiae vs. D. melanogaster (Table S2.1), D. melanogaster vs. D. melanogaster (Table S2.2), D. melanogaster vs. human (Table S2.3).

### RNAi knockdown experiments

RNAi knockdown experiments were performed in S2R+ cells as described previously [40]. We used the SnapDragon tool from the *Drosophila* RNAi Screening Center [41], [42] to design double stranded RNAs (dsRNA) for RNA interference (RNAi) analysis of the eight MFS type I transporters expressed in S2R+ cells (Table 1).

**Table 1:** *Drosophila* orthologs of yeast Pho84 and human SLC17A1–9.

Species	Ensembl Gene ID	Ensembl Transcript ID	Ensembl Protein ID	MGH-nomenclature	Associated Gene Name
S.cerevisiae	YML123C	YML123C	YML123C		pho84
D.melanogaster	FBgn0031307	FBtr0077958	FBpp0077623	MFS3	CG4726
D.melanogaster	FBgn0030452	FBtr0073724	FBpp0073555	MFS10	CG4330
D.melanogaster	FBgn0010497	FBtr0080292	FBpp0079876	MFS13	l(2)01810
D.melanogaster	FBgn0010651	FBtr0086635	FBpp0085817	MFS14	l(2)08717
D.melanogaster	FBgn0034392	FBtr0086636	FBpp0085818	MFS15	CG15094
D.melanogaster	FBgn0034611	FBtr0071590	FBpp0071516	MFS16	CG10069
D.melanogaster	FBgn0058263	FBtr0113848	FBpp0112571	MFS17	CG40263
D.melanogaster	FBgn0058263	FBtr0113848	FBpp0112571	MFS17	CG40263
D.melanogaster	FBgn0025684	FBtr0077456	FBpp0077146	MFS18	CG15438
D.melanogaster	FBgn0039886	FBtr0085870	FBpp0085229		CG2003
D.melanogaster	FBgn0039886	FBtr0085870	FBpp0085229		CG2003
D.melanogaster	FBgn0031645	FBtr0077391	FBpp0077083		CG3036
D.melanogaster	FBgn0031645	FBtr0077391	FBpp0077083		CG3036
D.melanogaster	FBgn0038099	FBtr0300278	FBpp0289506		CG7091
D.melanogaster	FBgn0016684	FBtr0087463	FBpp0086593	MFS2	NaPi-T
D.melanogaster	FBgn0033234	FBtr0088849	FBpp0087925	MFS12	CG8791
D.melanogaster	FBgn0034394	FBtr0086632	FBpp0085814		CG15096
D.melanogaster	FBgn0042126	FBtr0289986	FBpp0288424		CG18788
D.melanogaster	FBgn0024315	FBtr0087115	FBpp0086261	MFS8	Picot
D.melanogaster	FBgn0033048	FBtr0086033	FBpp0085369		CG7881
D.melanogaster	FBgn0028513	FBtr0080512	FBpp0080090		CG9254
D.melanogaster	FBgn0029727	FBtr0070715	FBpp0070683		CG6978
D.melanogaster	FBgn0031424	FBtr0077769	FBpp0077449	MFS11	VGlut
D.melanogaster	FBgn0034490	FBtr0086320	FBpp0085628		CG9864
D.melanogaster	FBgn0034782	FBtr0071892	FBpp0071803		CG12490
D.melanogaster	FBgn0034783	FBtr0071893	FBpp0071804		CG9825
D.melanogaster	FBgn0034784	FBtr0071895	FBpp0071806		CG9826
D.melanogaster	FBgn0034785	FBtr0071896	FBpp0071807		CG3649
D.melanogaster	FBgn0038799	FBtr0083888	FBpp0083296	MFS9	CG4288
D.melanogaster	FBgn0050265	FBtr0071891	FBpp0071802		CG30265
D.melanogaster	FBgn0050272	FBtr0071890	FBpp0071801	MFS1	CG30272
H.sapiens	ENSG00000124568	ENST00000244527	ENSP00000244527		SLC17A1
H.sapiens	ENSG00000112337	ENST00000377850	ENSP00000367081		SLC17A2
H.sapiens	ENSG00000124564	ENST00000360657	ENSP00000353873		SLC17A3
H.sapiens	ENSG00000146039	ENST00000377905	ENSP00000367137		SLC17A4
H.sapiens	ENSG00000119899	ENST00000355773	ENSP00000348019		SLC17A5
H.sapiens	ENSG00000091664	ENST00000263160	ENSP00000263160		SLC17A6
H.sapiens	ENSG00000104888	ENST00000221485	ENSP00000221485		SLC17A7
H.sapiens	ENSG00000179520	ENST00000323346	ENSP00000316909		SLC17A8
H.sapiens	ENSG00000101194	ENST00000370351	ENSP00000359376		SLC17A9
H.sapiens	ENSG00000160190	ENST00000352133	ENSP00000344648		SLC37A1
H.sapiens	ENSG00000134955	ENST00000308074	ENSP00000311833		SLC37A2
H.sapiens	ENSG00000157800	ENST00000326232	ENSP00000321498		SLC37A3
H.sapiens	ENSG00000137700	ENST00000330775	ENSP00000339048		SLC37A4

Transporter protein IDs used for BLAST and Bayes phylogenetic analysis.

dsRNA was synthesized from PCR templates using the T7 Megascript kit (Ambion). See Table S1 for the primer sequences used. Following TAE-2% agarose gel electrophoresis and densitometric quantification at 260 nm (Nano-drop 8000, Fisher Scientific) for quality control, 200.000 S2R+ cells/well in 48-multiwell plates were incubated with 3 ug dsRNA per well in 250 ul serum-, phosphate- and antibiotic-free medium supplemented with 10 mM HEPES (pH 7.4) for 45 min. at 25 C. Transfection was stopped by the addition of 250 ul phosphate-free medium containing 20% heat-inactivated FBS, 2× penicillin/streptomycin and 10 mM HEPES (pH 7.4). After culture for three days cells were challenged with 10 mM sodium-phosphate (pH7.4) or 25 ug/ml Insulin for 3 min. Lysates were analyzed by Western analysis as described above. For quantitative RT-PCR analysis, lysates were prepared using 300 ul RLT-PLUS per well according to the manufacturer's instructions for RNeasy-micro PLUS kit (Qiagen). Following reverse transcription using the Omni-script kit (Qiagen), quantitative PCR using the Cybr Green kit (Qiagen) was performed using intron-overlapping primers, which had been chosen so to not overlap the dsRNA target sequences (see Table S1 for primer sequences). To calculate efficiency of knockdown, target mRNA expression corrected for the actin mRNA expression of cells treated with target RNAi was compared to target mRNA expression corrected for actin mRNA expression of cells treated with RNAi targeting luciferase, a gene that is not expressed in fly cells, and thus serves to control for non-specific RNAi effects. qRT-PCR of dPit mRNA was unaffected by RNA-mediated knockdown of MFS10 and MFS13 and vice versa. To exclude off-target effects of the dsRNA affecting phosphate-induced MAPK independent of the transporters under investigation we furthermore showed that several independent dsRNAs targeting MFS10, 13 and Pit1 have similar effects.

### Phosphate-uptake studies

Plasmids encoding full-length cDNAs of *FBgn0010497*, *FBgn0030452*, and *FBgn0260795* were obtained from the *Drosophila* Genome Resource Center (DGRC, https://dgrc.cgb.indiana.edu/). Flybase (http://flybase.org/) notes only one transcript and protein for each transporter genes and nucleotide sequence analysis was used to independently confirm presence of initiation codon and poly-adenylation signal in the cDNAs obtained from the DGRC prior to preparation of cRNA for expression in *Xenopus* oocytes using the mMessage Machine T7 and polyA-tailing kits (Ambion), and PCR-based templates generated using a sense primer containing the T7 promotor sequence (for primer sequences see Table S1). Full length of cRNA transcripts was confirmed using denaturing gel electrophoresis, and thus it is unlikely that non-functional splice variants or incomplete proteins were expressed.

For phosphate-uptake experiments, *Xenopus* oocytes were harvested from female frogs by C-section, de-folliculated and injected with 50 ng capped and poly-adenylated RNA in 100 nl per oocyte, prepared with the mMessage Machine kit (Ambion) as previously described [43]. Following incubation in ND96+++ buffer (NaCl 0.192 M; KCl 4 mM; HEPES pH 7.4, 20 mM; CaCl2 1.8 mM; MgCl2 1 mM; 1× penicillin/streptomycin) for three days at 18 C to permit protein expression, phosphate uptake was measured in ND100 (100 mM NaCl, 2 mM KCl, 1.8 mM CaCl2, 1 mM MgCl2, 1 mM Pi, 10 mM Hepes-Tris (pH 7.4), supplemented with [33P]-orthophosphoric acid (Perkin Elmer) (final specific activity, 5–50 mCi/mmol) at room temperature for 60 min., followed by four washes in ND0+2 mM Pi, lysis and detection of single-oocyte uptake using a scintillation counter. For sodium-free conditions 100 mM choline-chloride was substituted for 100 mM sodium-chloride to obtain ND0.

### Fly culture and crosses

Standard fly culture was performed at 25°C on 17 g/l yeast, 9.8 g/l soy flour, 71 g/l corn meal, 5.6 g/l agar, 5.6 g/l malt, 75 ml/l corn syrup, 4 ml/l propionic acid and 250 mg/l tegosept (Spectrum M1187). This medium was supplemented with 30 mM sodium-phosphate (pH6.0), sodium-sulfate (pH 6.0), 10 mM phosphonoformic acid (Sigma P6801), or 1% sevelamer (gift from Dr. Yves Sabbagh, Genzyme, Inc.).

P-element insertions and deficiency mutants targeting the genomic locus of MFS13 (*FBgn0010497*) were obtained from the Bloomington *Drosophila* Stock Center (BDSC, http://flystocks.bio.indiana.edu/), and the Exelexis collection (https://drosophila.med.harvard.edu/): *P{PZ}l(2)01810^01810^/CyO; ry506* (Bloomington stock 11076), *y1 w67c23 P{wHy}l(2)01810^DG29108^* (Bloomington stock 20492), y1 w*; Mi{MIC}l(2)01810^MI00602^, P{XP}l(2)01810^d06403^, PBac{RB}l(2)01810^e02547^, w1118; Df(2L)BSC826/SM6a (Bloominton stock 27900), *w1118; Df(2L)BSC323/CyO* (Bloomington stock 24348) (for genomic location of these insertions see Figure S1A and B).

While four P-element insertions were homozygous viable, *P{PZ}l(2)01810^01810^ cn1* and the deficiencies were lethal. To examine, whether lack of viability of these stocks is related to homozygous loss of *MFS13*, we generated flies of the genotypes *P{PZ}l(2)01810^01810^/Df(2L)BSC826* and *P{PZ}l(2)01810^01810^/Df(2L)BSC323,* which were viable. Loss of *MFS13* in these heterozygous flies was confirmed by quantitative RT-PCR. Briefly, 5 flies were collected for total RNA preparation using Trizol reagent (Invitrogen). cDNA was synthesized using the Omniscript cDNA reverse transcription kit (Qiagen). The levels of mRNA for different genes were measured by using SYBR-GREEN QuantiTect (Qiagen) on a StepOnePlus real time PCR system (Applied Biosystems). For these experiments RpL32 (*FBgn0002626*) was used for normalization, which, unlike actin5C, is not influenced by culture temperature of the flies [44]. The primers used are listed in Table S1.

### Statistical analysis

Assay variability was generally less than 10% for Westernblot, qPCR and phosphate uptake experiments. Means±SEM of at least three independent experiments performed in duplicate are shown.

## Results

### Phosphate activates MAPK in S2R+ cells in a time and dose-dependent fashion

**Figure 1:**
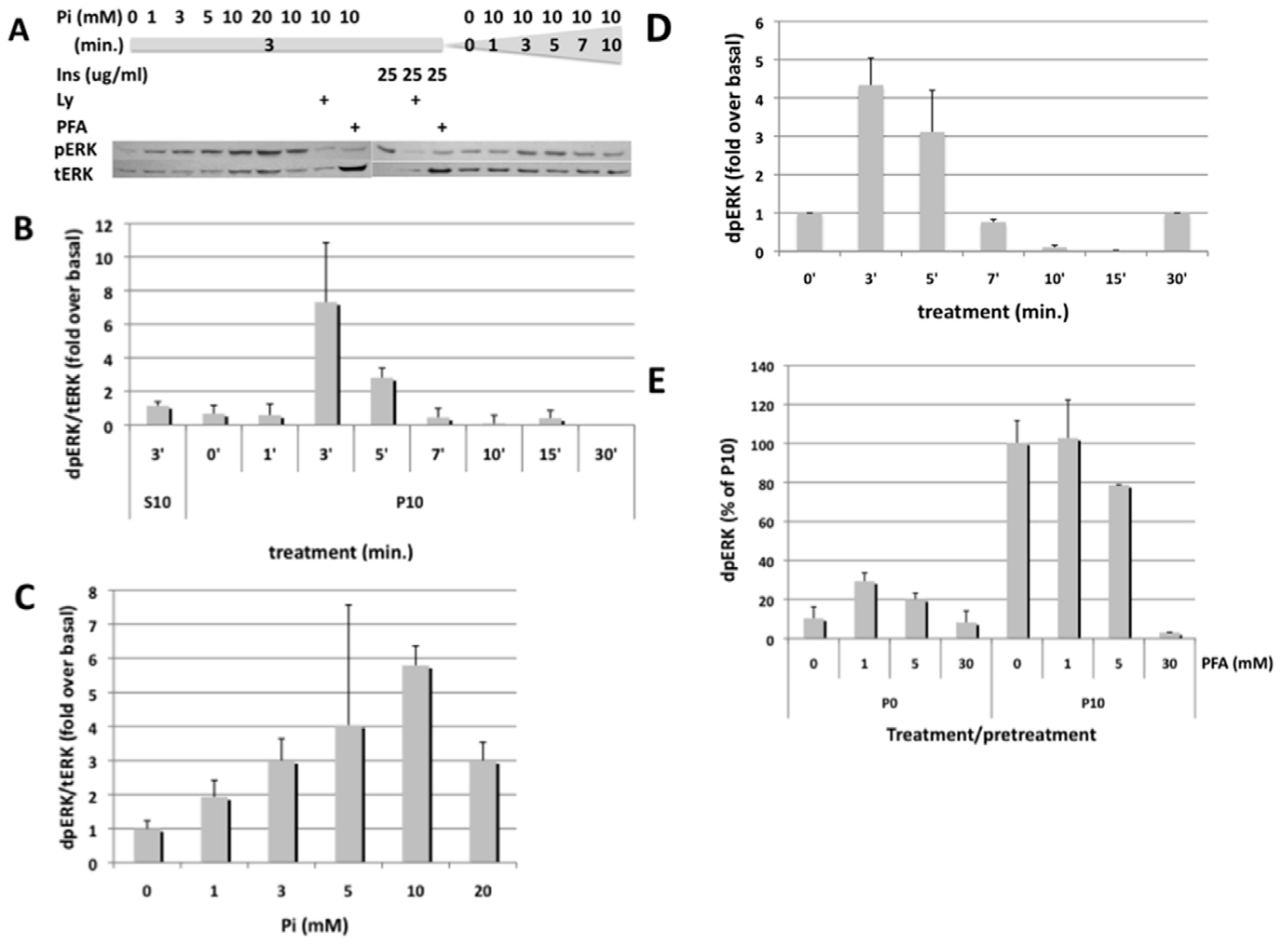
Western blot analysis of phosphate-induced MAPK in S2R+ and Kc167 cells. **A:** Dose response and time course of phosphate-activated MAPK in S2R+ cells. **B:** Dose response of phosphate-activated MAPK in S2R+ cells. **C:** Time course of phosphate-activated MAPK in S2R+ cells. **D:** Time course of phosphate-activated MAPK in Kc167 cells. **E:** Effect of PFA on activation of MAPK in S2R+ cells Shown are one representative Western blot autoradiogram (A), or pooled densitometic data of at least three independent Western blot experiments (B–E). Abbreviations: Pi = inorganic phosphate, Ins = human insulin, Ly = Ly294002 (PI3K-inhibitor, 50 uM), PFA = phosphonoformic acid (30 mM, unless otherwise noted), S10 = sodium sulfate (10 mM), P10 = sodium phosphate (10 mM).

To establish an assay for phosphate sensing in *Drosophila*, we investigated whether, as observed in mammalian cells, phosphate can activate MAPK in *Drosophila* cells. *Drosophila* S2R+ cells were exposed to 10 mM sodium-phosphate buffer (pH7.4) and a phospho-specific ERK antibody was used to detect MAPK response (Fig. 1A). Phosphate activates MAPK rapidly within 3 min. and desensitizes over the course of 15 min (Fig. 1B). Activation is dose-dependent, and reaches a maximum at 10 mM (Fig. 1C). Activation of MAPK is not seen with an iso-osmolar stimulus of 10 mM sodium-sulfate (Fig. 1B). The time course of activation by phosphate is similar to activation of MAPK by 25 ug/ml insulin, but when compared to phosphate, activation of MAPK by insulin appears to be more sustained and returns back to baseline after 30–60 min. (data not shown). Long-term exposure to 10 mM phosphate or insulin over 24 hrs does not lead to significant activation of MAPK above baseline (data not shown). Similar time-dependent activation of MAPK is seen in a second *Drosophila* hemocyte-like cell line, Kc167 (Fig. 1D). Different, however, from S2R+ cells, activation of MAPK is followed by suppression below baseline after 10 and 15 min, with return to baseline after 30 min.

Addition of phosphonoformic acid (PFA) blocks activation of MAPK by phosphate in mammalian cell lines [29], [30], indicating that binding or cellular uptake of phosphate is required for the activation of MAPK. Similarly, exposure to PFA for 60 min. prior to stimulation with 10 mM phosphate blocked activation of MAPK in S2R+ cells, although higher doses were required (30 mM) when compared to what is effective in mammalian cell lines (5–10 mM, [29], [30])(Fig. 1E). Importantly, PFA blocks phosphate- but not insulin-induced MAPK in S2R+ cells, indicating that phosphate activates MAPK using a different signaling pathway. Phosphate, furthermore, is unable to induce phosphorylation of AKT in S2R+ cells (data not shown).

### *Drosophila* cell lines express orthologs of mammalian type I and type III phosphate transporters

**Figure 2:**
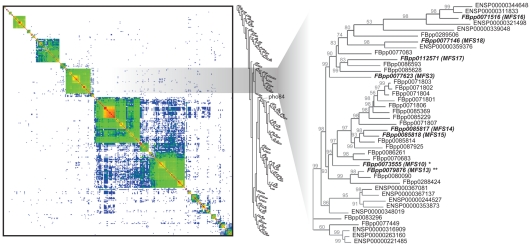
Blast and Bayes analysis of MFS transporters. Heatmap of pairwise BLAST bit scores for all known yeast, *Drosophila* and human proteins containing the MFS protein domain PF07690 [38] (left panel) sorted by a hierarchical clustering (middle panel). Bayesian phylogenetic reconstruction (dendogram) was used to identify 29 fly orthologs that are most closely related to yeast *Pho84* (YML123C) and human *SLC17A1–9*. Posterior probabilities are indicated above each branch. Fly transporters found to be expressed in S2R+ cells are shown in bold/italic script.

To characterize the *Drosophila* phosphate transporters involved in MAPK activation, we determined which orthologs of mammalian type I and type III phosphate transporters are expressed in *Drosophila* S2R+ cells. The *Drosophila* genome contains orthologs of mammalian type I and type III phosphate transporters, but lacks orthologs of the mammalian type II transporters [22]. While there is only one *Drosophila* type III transporter ortholog, dPit (*FBgn0260795*), type I phosphate transporters belong to the MFS and share the protein domain PF07690, which is present in 77 yeast proteins, 219 *Drosophila* proteins, and 229 human proteins. We used BLAST followed by Bayes phylogenetic analysis to identify 29 fly orthologs that are most closely related to yeast *Pho84* and human *SLC17A1–9* (Fig. 2, Table 1, Tables S2.1–3).

**Figure 3:**
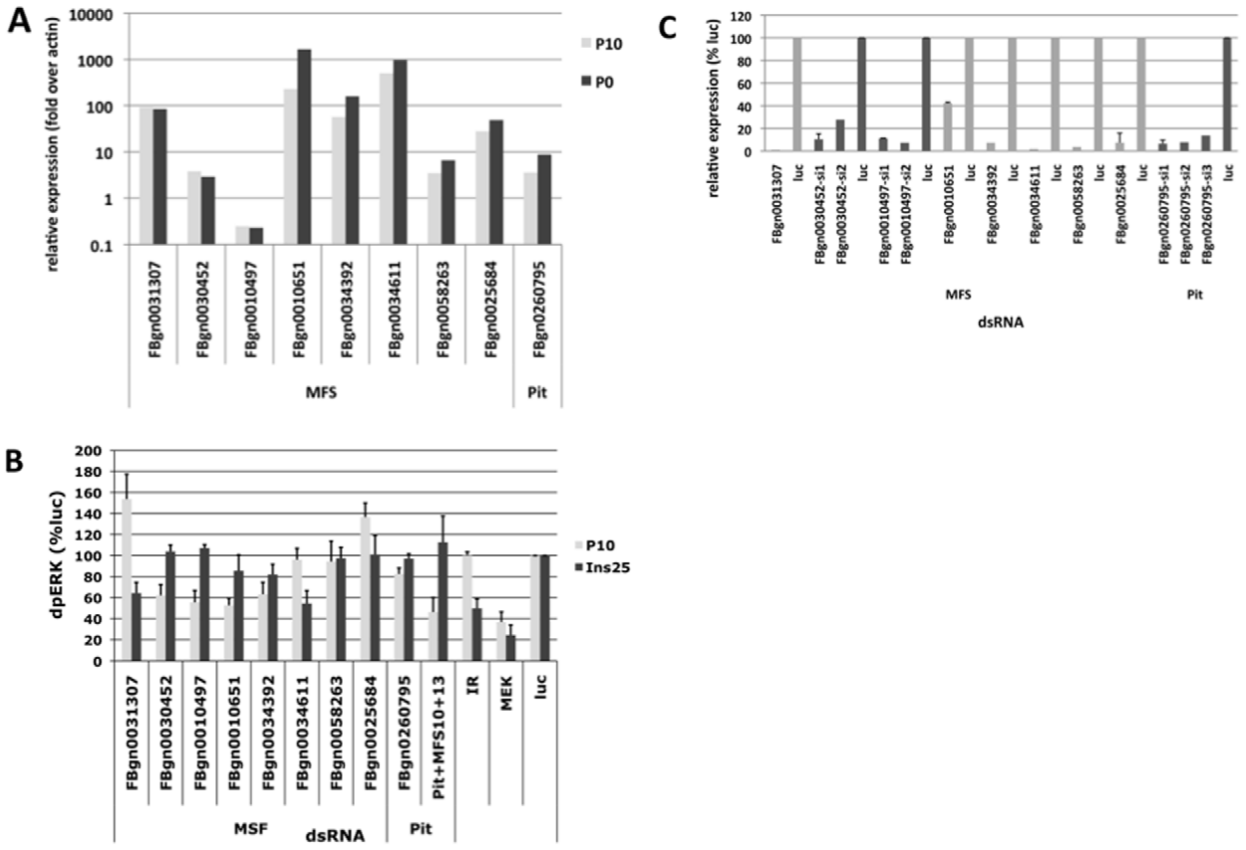
Effect of RNAi knockdown of MFS transporters and *dPit* on MAPK. **A**: mRNA expression of MFS and *Pit* transporters in S2R+ cells. Data of three replicate experiments are shown as mean±SEM expression relative to actin 5 C. **B**: Effect of RNAi knockdown of MFS transporters and *dPit* on MAPK. Data of three replicate experiments are shown as mean±SEM relative to cells transfected with dsRNA targeting lucifierase (luc). **C**: RNAi knockdown efficiency. To calculate efficiency of knockdown, parallel wells prepared for pERK1/2 Western analysis above (Fig. 2B) were used for total RNA extraction and quantitative RT-PCR. Shown are mean±SEM of three replicate experiments after expression was corrected for actin 5 C mRNA. Cells treated with dsRNA targeting luciferase are set 100% for each specific primer pair.

Eight of these transporters are expressed in S2R+ cells when checked against publically available cell-specific RNA expression profiles using high-density genome tiling microarrays from ModENCODE [45], and Affymetrix and Flychip *Drosophila* expression array data from FLIGHT [46] (see Table S3). The expression profile of these eight MFS transporters was similar in 17 *Drosophila* cell lines including S2R+ and Kc167 cells, consistent with a universal role in phosphate-sensing (see Table S4). Expression of the eight transporters was confirmed using qRT-PCR of total RNA extracted from S2R+ cells (Fig. 3A). Relative expression levels were tested with several independent primer sets for qRT-PCR and found to be within one order of magnitude of the expression of *Drosophila* actin 5 C. Modest up-regulation of the expression of some transporters was observed, when S2R+ cells were cultured for three days in the absence of phosphate (Fig. 3A).

The sequence alignment of the eight expressed fly transporters shows 7.6–12.9% amino acid identity to *pho84,* compared to 12.2–45.2% amino acid identity among each other (Fig. S2A), with higher degree identity seen in hydrophobic, predicted membrane-spanning regions of these transporters (18.9–27.5% to pho84 and 19.1–51.1% among the fly transporters (Fig. S2B and S2C).

### RNAi knockdown of MSF transporters blocks phosphate- but not insulin-induced MAPK in S2R+ cells

siRNA-mediated knockdown of the *Pit1* sodium-phosphate co-transporter blocks activation of MAPK by phosphate in human embryonic kidney (HEK293) cells [27] indicating that this type III sodium-phosphate co-transporter [19] is required for the activation of MAPK in this cell line. However, RNAi knockdown of the *dPit* only reduced phosphate-induced MAPK by 20% in S2R+ cells (Fig. 3B). Findings were similar with three independent dsRNAs and quantitative RT-PCR confirmed that the *Pit1* mRNA level was reduced 100-fold when compared to baseline, i.e. cells transfected with dsRNA targeting luciferase, a gene not expressed in S2R+ cells and thus serving as a control for non-specific RNAi effects (Fig. 3C).

Individual knockdown of two of the eight expressed MFS transporters, MFS10 and MFS13 (encoded by *FBgn0030452* and *FBgn0010497,* respectively) resulted in 40% reduction of phosphate-induced MAPK, which exceeds the effect seen by knockdown of *dPit* (Fig. 3B). Knockdown of these MFS transporters was specific for phosphate, since insulin continued to be able to stimulate MAPK. These results were reproducible by two independent sets of dsRNAs targeting MFS10 and MFS13). Furthermore, knockdown of all three transporters was additive and resulted in 60% reduction of phosphate-induced MAPK. Conversely, RNAi targeting the insulin receptor blocked insulin-induced MAPK, but not phosphate-induced MAPK. Finally, RNAi-knockdown of the upstream kinase, MEK, blocked stimulation of MAPK in response to both stimuli, indicating that MAPK phosphorylation by phosphate is mediated by MEK.

Interestingly, one transporter (encoded by *FBgn0031307*) appears to be a specific negative regulator of phosphate-induced MAPK. Further, while insulin-induced MAPK was unaffected, two transporters (encoded by *FBgn0010651* and *FBgn0034392*) were positive regulators of both phosphate- and insulin-induced MAPK, while knockdown of three transporters (encoded by *FBgn0034611, FBgn0058263*, and *FBgn0025684*) had no significant effect on phosphate-induced MAPK.

### MFS13 (*FBgn0010497*) mediates Na-dependent phosphate-uptake when expressed in *Xenopus* oocytes

To test whether MFS10 and MFS13 facilitate cellular phosphate uptake, we injected capped and poly-adenylated sense RNA encoding these transporters into *Xenopus* oocytes. Following injection of 50 ng/oocyte and culture at 18 C for three days to allow for expression of the transporter protein in the oocyte plasma membranes, we performed a radioactive-phosphate uptake experiment in the absence or presence of sodium and PFA at pH5.5, 7.4, and 8.5.

**Figure 4:**
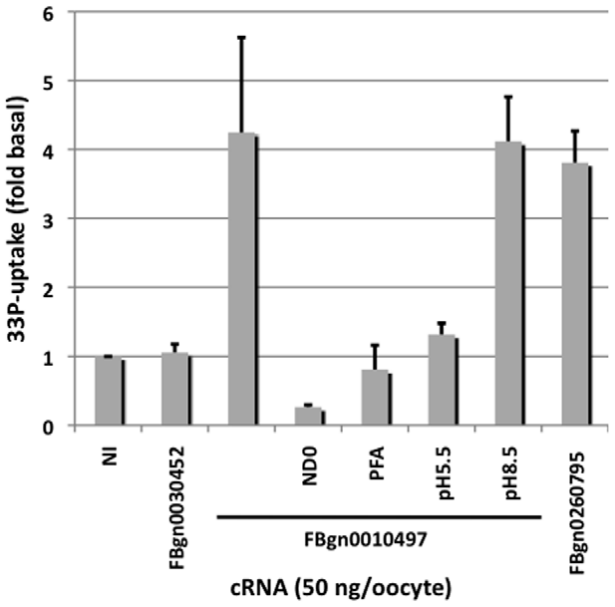
Phosphate transport after expression of MFS and *dPit* transporters in X. oocytes. Phosphate uptake of *Xenopus* oocytes injected with capped RNA encoding MFS10 (FBgn0030452), MFS13 (FBgn0010497), and *dPit* (*FBgn0260795*), was measured in ND100+^33^P, or ND0+^33^P, in the presence or absence of 5 mM PFA at pH7.4 or at pH5.5 or 8.5 where indicated. ^33^P-uptake is expressed in multiples over basal seen with non-injected oocytes as mean±SEM from at least three different batches of oocytes (n = 10/bath).

MFS13 showed significant uptake of phosphate, while no significant uptake was seen when expressing MFS10. Uptake mediated by MFS13 was similar in magnitude to that seen with *dPit* but 10% when compared to that seen with oocytes expressing human *SLC34A3* (*NaPi-IIc*)(data not shown). Radioactive phosphate uptake was dependent on sodium and blocked by PFA or low pH, while transport was maximal at physiological pH 7.4 and at pH 8.5 (Fig. 4). Altogether, these results indicate that MSF13, but not MSF10, mediates uptake of [^33^P]-orthophosphate in a sodium-dependent fashion.

### Phosphonoformic acid and sevelamer impair larval development in fly

A search in FlyAtlas [47] reveals that MFS10 (*FBgn0030452*) mRNA is expressed highest in the male accessory gland, two-fold enriched in brain and four-fold enriched in the Malpighian tubule, the renal tubule equivalent in fly, when compared to whole fly expression. MFS13 *(FBgn0010497)* mRNA is expressed highest in the crop, midgut, Malpighian tubule, and hindgut, where it is three-fold enriched when compared to whole fly (Fig. S3). No entry is found for *dPit*.

To explore the role of phosphate during larval development of *Drosophila* we cultured wild-type flies in 0.5% sevelamer to inhibit absorption of dietary phosphate [48] and 1 mM PFA to block phosphate transport into cells [49]. This treatment delayed embryonic and larval development (Fig. S4A and B). The effect of sevelamer and PFA was reversed by addition of 30 mM sodium phosphate.

To further evaluate the role of MFS13 *in vivo*, we obtained a P-element insertion in MFS13 (P{PZ}l(2)01810^01810^) that was viable over two deficiencies of the region (Df(2L)BSC826, Df(2L)BSC323). qPCR analysis of adult flies of the genotype *P{PZ}l(2)01810^01810^/Df(2L)BSC826* or *P{PZ}l(2)01810^01810^/Df(2L)BSC323* revealed that MFS3 expression is most likely completely absent, suggesting that MSF13 mediates phosphate transport together with other transporter(s) (Fig. S1C).

## Discussion

In this study, we show that activation of MAPK is part of the down-stream events stimulated when two *Drosophila* hemocyte-like cell lines, S2R+ and Kc167, are exposed to phosphate. Just like in mammalian cell lines, we furthermore found that PFA blocks phosphate induced MAPK in S2R+ *Drosophila* cells. Activation of MAPK by phosphate, which thus far has only been shown in mammalian cell lines (reviewed in: [20], [21]), consequently appears to be evolutionarily conserved.

Activation of the MAPK pathway by phosphate in metazoan species is likely relevant for cellular functions as has been shown for the regulation of RANK/RANK-L signaling [50], mRNA expression of bone matrix proteins osteopontin [24], and matrix gla protein [51] or down-regulation of type III transporters *Pit1* and *Pit2* [52], all of which are blocked by UO126, an inhibitor of the upstream MAPK-kinase MEK. Yet, it is poorly understood, whether phosphate needs to enter metazoan cells to stimulate intracellular signaling events as suggested by the inhibitory action of PFA, or whether it binds and activates a cell surface receptor.

In yeast the major facilitator superfamily transporters *Pho84* and the type III transporter *Pho 89* have been implicated in phosphate-sensing in yeast [16]. Recent evidence suggests that the mammalian ortholog of *Pho89, Pit1*, mediates cellular effects of phosphate, however, we found in S2R+ cells that knockdown of the fly ortholog *dPit* only reduced activation of MAPK by phosphate by 20% when compared to control, while it reduced *dPit* mRNA by more than 90%. Since orthologs of the type II co-transporters are absent from the *Drosophila* genome, we therefore postulated that a type I co-transporter ortholog related to *Pho84* may be involved in phosphate sensing in *Drosophila* S2R+ cells. Despite sequence divergence and size of this transporter family we were able to identify eight fly *Pho84* candidates based on sequence homology to the human MFS transporters *SLC17A1–9*, and expression in our cell line. These eight transporters are highly expressed in a number of other fly cell lines as shown in Table S4, supportive of their universal role for phosphate-sensing. Evaluation of these eight MFS members using phosphate-induced MAPK as readout provides evidence that four *Drosophila* type I (MFS) transporters are positive regulators, while one transporter is a negative regulator of phosphate-induced MAPK. Three of these five transporters specifically affect phosphate, while insulin-induced MAPK was unaffected. We decided to further investigate the two positive and specific regulators MFS10 and MFS13 (encoded by *FBgn0030452* and *FBgn0010497*), which are required for the activation of MAPK by phosphate in S2R+ cells. Further evaluation after expression in *Xenopus* oocytes indicates that one of these two transporters (MFS13, encoded by *FBgn0010497*) shows significant phosphate conductance, which is comparable in magnitude to that seen with *dPit*. Consistent with the mechanism of transport known for human *SLC17A1–9*, this phosphate conductance is sodium-dependent and inhibited by PFA or low pH. Our findings therefore provide first evidence for the presence of multiple *Pho84* orthologs in a multicellular organism, which along with the *Pho89* ortholog *dPit* are involved in phosphate-sensing. The sequence alignment highlights conserved domains and residues which may be involved in these functions (Fig. S2C).

Since 5 mM PFA is sufficient to inhibit the MFS13 transporter after expression in X. oocytes, lower potency of PFA on MFS10, dPit or possibly other transporters may explain the high concentration of 30 mM PFA is needed to block phosphate induced MAPK in S2R+ cells.

Loss of *Pho84* reduces proliferation and survival in yeast, which can be rescued by over-expressing the related phosphate transporter *Pho89* [16], suggesting that members of different superfamilies permit cellular uptake of phosphate in yeast that then is sensed intracellularly. However, the fact that overexpression of a phosphate-transport deficient *Pho84* variant can rescue regulation of the extracellular alkaline phosphatase Pho5 by phosphate in *Pho84* deficient strains, while overexpression of *Pho89* is ineffective, suggests, that binding of extracellular phosphate alone may be sufficient, at least for some down-stream effects of phosphate in yeast [16]. Since multiple transporter are involved in S2R+, our findings support the possibility that cellular uptake of phosphate is required, and that also in metazoan cells intracellular phosphate is what is sensed and what leads to activation of MAPK.

This study has several limitations that require future investigation: only 29 out of 219 known *Drosophila pho84* orthologs were examined and it is possible that other orthologs are expressed and involved in phosphate-induced MAPK in S2R+ cells. Phosphate transport data shown here are qualitative in nature and future experiments have to include quantification of surface expression of the fly transporters. Since transport for phosphate by MFS13 and *dPit* was in our hands less efficient when compared to the human type II sodium-phosphate co-transporter NaPi-IIc (data not shown), and we were unable to show phosphate-conductance for the second type I transporter MFS10 (encoded by *FBgn0030452*) it is possible that extracellular binding of phosphate to these transporters leads to activation of intracellular events independent of phosphate-uptake. Based on studies in mammalian cells, it is possible, that *Pit1* is the sole functional paralog of yeast *Pho84* and *Pho89* in higher species [17], [19]. Indeed, targeted deletion, hypomorphic and overexpression mutants of *Pit1* support a fundamental role of this transporter in liver growth and phosphate homeostasis of mice [32], [33], [34]. However, additional mechanisms for phosphate-sensing possibly involving *Pho84* orthologs may exist since *Pit1* null mice exhibit normal embryonic development and morphogenesis. Consistent with an important role for phosphate in metabolism and endocrine regulation we found that PFA and sevelamer impair larval development of *Drosophila*. However, just like in mice we also found that deletion of MFS13 is compatible with larval development and metamorphosis of flies indicating that loss of a single transporter can be compensated by others *in vivo.*

In conclusion, our findings suggest that activation of MAPK by phosphate is evolutionarily conserved from fly to man. MFS transporters mediate cellular effects of phosphate in fly S2R+ cells along with *dPit*, which may be relevant for higher species and humans. Further studies are required to better understand the role of these transporters in *Drosophila* phosphate-homeostasis.

## Supporting Information

Figure S1 **P-element and deficiency stocks for MFS13 (** ***l(2)01810*** **,** ***FBgn0010497*** **).** The insertion sites of the P-elements obtained from flybase (www.flybase.org) is shown in **A,** the location of available chromosome 2 deficiency mutants surrounding the genetic locus and including *FBgn0010497* is shown in **B**. qRT-PCR to confirm complete loss of MFS13 transcripts in *P{PZ}l(2)01810^01810^/Df(2L)BSC826* (11076/27900) or *P{PZ}l(2)01810^01810^/Df(2L)BSC323* (11076/24348) adult flies when compared to heterozygous stocks and wild-type flies (CTRL) (**C**).
(TIF)

Figure S2 **Sequence comparison between ph84 and MFS transporters expressed in S2R+ cells.** **A:** Global alignment. Amino acid sequence identity in % between the sequence shown in column and row (alignment length in brackets). **B:** Local alignment. Amino acid sequence identity in % between sequence shown in column and row (alignment length in brackets). **C:** Clustal W alignment of fly transporters expressed in S2R+ cells along with Pho84 using Jalview (http://www.jalview.org/download.html).
(PDF)

Figure S3 **FlyAtlas tissue distribution of MFS10 (FBgn0030452) and MFS13 (FBgn0010497).** Using FlyAtlas [47](http://flyatlas.org/) mRNA expression of MFS10, and MFS13 (encoded by *FBgn0030452*, *FBgn0010497,* respectively) is shown for various larval and adult fly tissues.
(TIF)

Figure S4 **Phosphonoformic acid and sevelamer impair larval development.** Yellow white flies were cultured on standard medium at 25°C. This medium was supplemented with 30 mM sodium-phosphate (pH6.0)(P30), 1 mM phosphonoformic acid (PFA), or 0.5% sevelamer (Sev) or in combinations thereof. Number of larvae emerged from the medium over time are shown.
(TIF)

Table S1 **Primer sequences used for dsRNA synthesis, cRNA synthesis and qRT-PCR.** Primer sequences are displayed 5′ to 3′ and include the T7-RNA-polymerase promotor when used to generate PCR templates for dsRNA or cRNA sythesis.
(XLS)

Table S2 **Tables of all blast hits S. cerevisiae vs. D. melanogaster (S2.1), D. melanogaster vs. D. melanogaster (S2.2), D. melanogaster vs. human (S2.3) with the following format.** 1. Query ID (Ensembl). 2. Subject ID (Ensembl). 3. % identity between query and subject, 4. alignment length between query and subject, 5. mismatches between query and subject, 6. gap openings between query and subject, 7. query start, 8. query end, 9. subject start, 10. subject end, 11. e-value, 12. bit score. 13. query family ID. 14. subject family ID.
(XLS)

Table S3 **MFS expression in S2R+ cells.** Cell-specific RNA expression profiling for S2R+ cells using high-density genome tiling microarrays (next generation RNAseq technology) was obtained from ModENCODE [45], and using Affymetrix Flychip *Drosophila* expression array technology was obtained from FLIGHT [46]. Expression cut-off's are given in brackets and expressed genes are high-lighted in green.
(XLS)

Table S4 **Expression data for** ***Drosophila*** **MFS transporters in S2R+ cells.** Cell-specific RNA expression profiling of 17 *Drosophila* cell lines using high-density genome tiling microarrays (next generation RNAseq technology) was obtained from ModENCODE [45], and using Affymetrix Flychip *Drosophila* expression array technology was obtained from FLIGHT [46]. Expressed genes are highlighted in green (or blue for modest expression).
(XLS)
