# Supplementary figures and images for: Roles of Major Facilitator Superfamily Transporters in Phosphate Response in Drosophila
Source: PLoS One. 2012 Feb 16;7(2):e31730. doi: 10.1371/journal.pone.0031730 (PMC3280997; doi:10.1371/journal.pone.0031730)

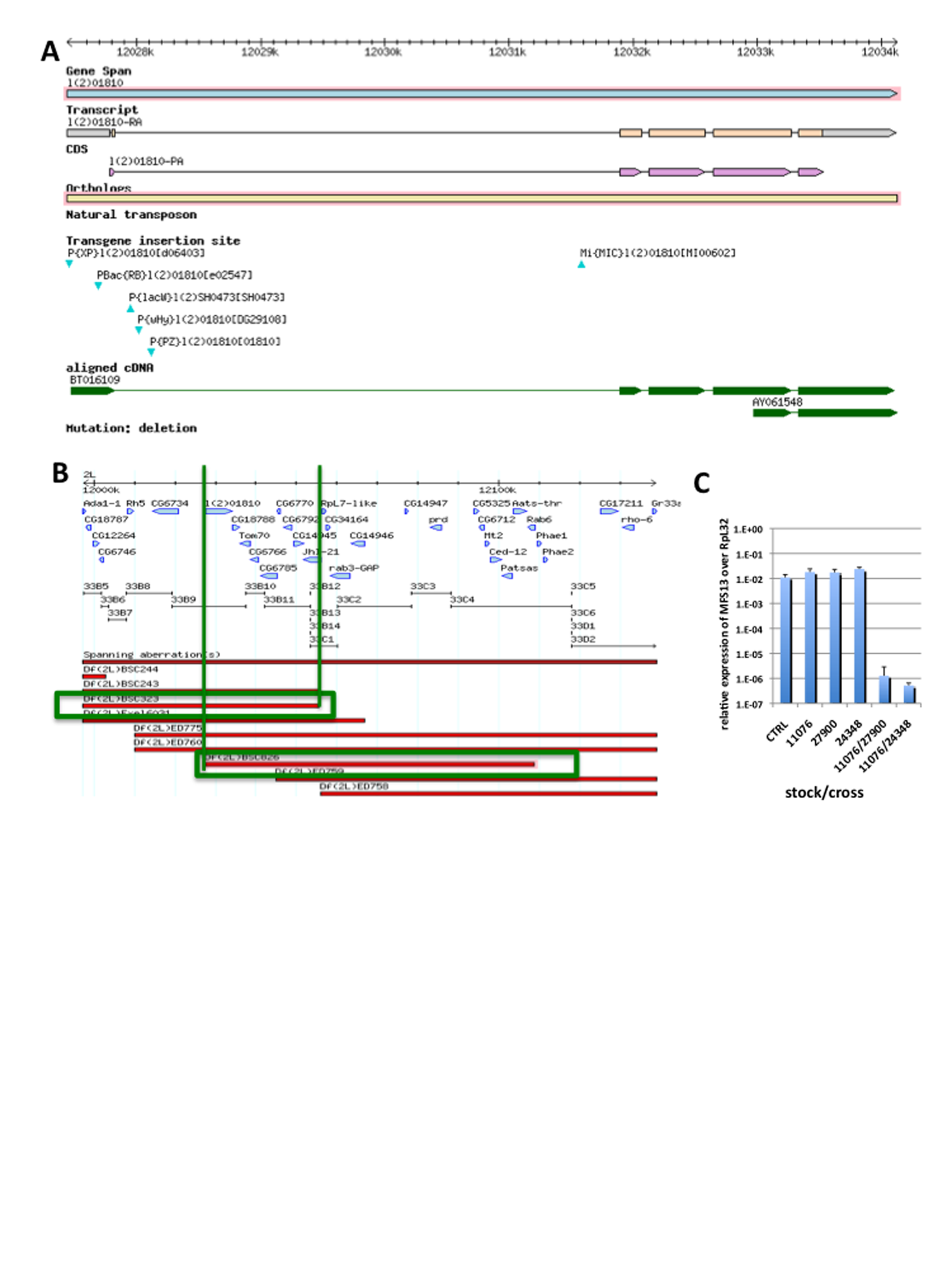

Supplement: Figure S1 — P-element and deficiency stocks for MFS13 ( l(2)01810 , FBgn0010497 ). The insertion sites of the P-elements obtained from flybase (www.flybase.org) is shown in A, the location of available chromosome 2 deficiency mutants surrounding the genetic locus and including FBgn0010497 is shown in B. qRT-PCR to confirm complete loss of MFS13 transcripts in P{PZ}l(2)0181001810/Df(2L)BSC826 (11076/27900) or P{PZ}l(2)0181001810/Df(2L)BSC323 (11076/24348) adult flies when compared to heterozygous stocks and wild-type flies (CTRL) (C). (TIF) [file pone.0031730.s001.tif]

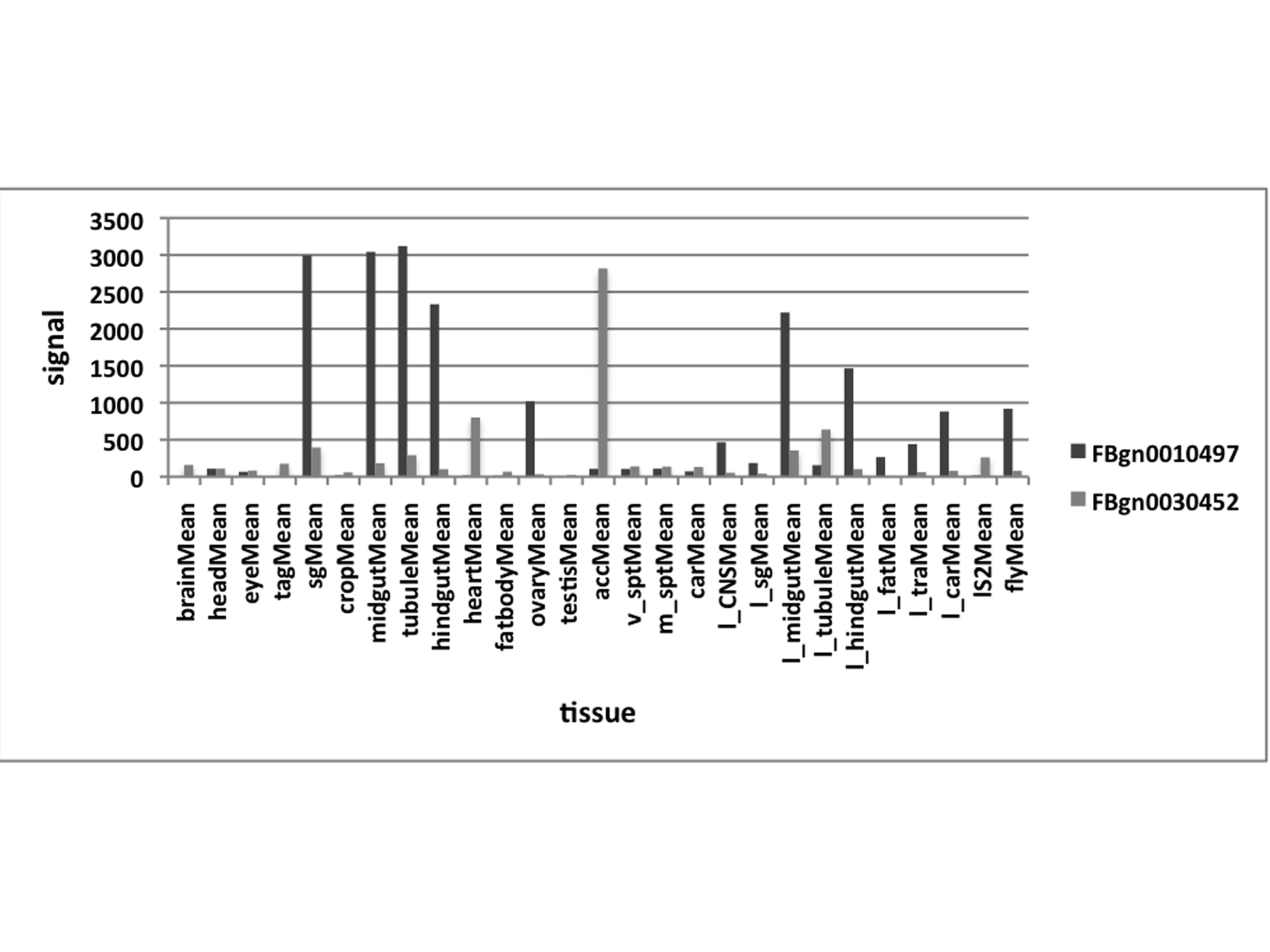

Supplement: Figure S3 — FlyAtlas tissue distribution of MFS10 (FBgn0030452) and MFS13 (FBgn0010497). Using FlyAtlas [47](http://flyatlas.org/) mRNA expression of MFS10, and MFS13 (encoded by FBgn0030452, FBgn0010497, respectively) is shown for various larval and adult fly tissues. (TIF) [file pone.0031730.s003.tif]

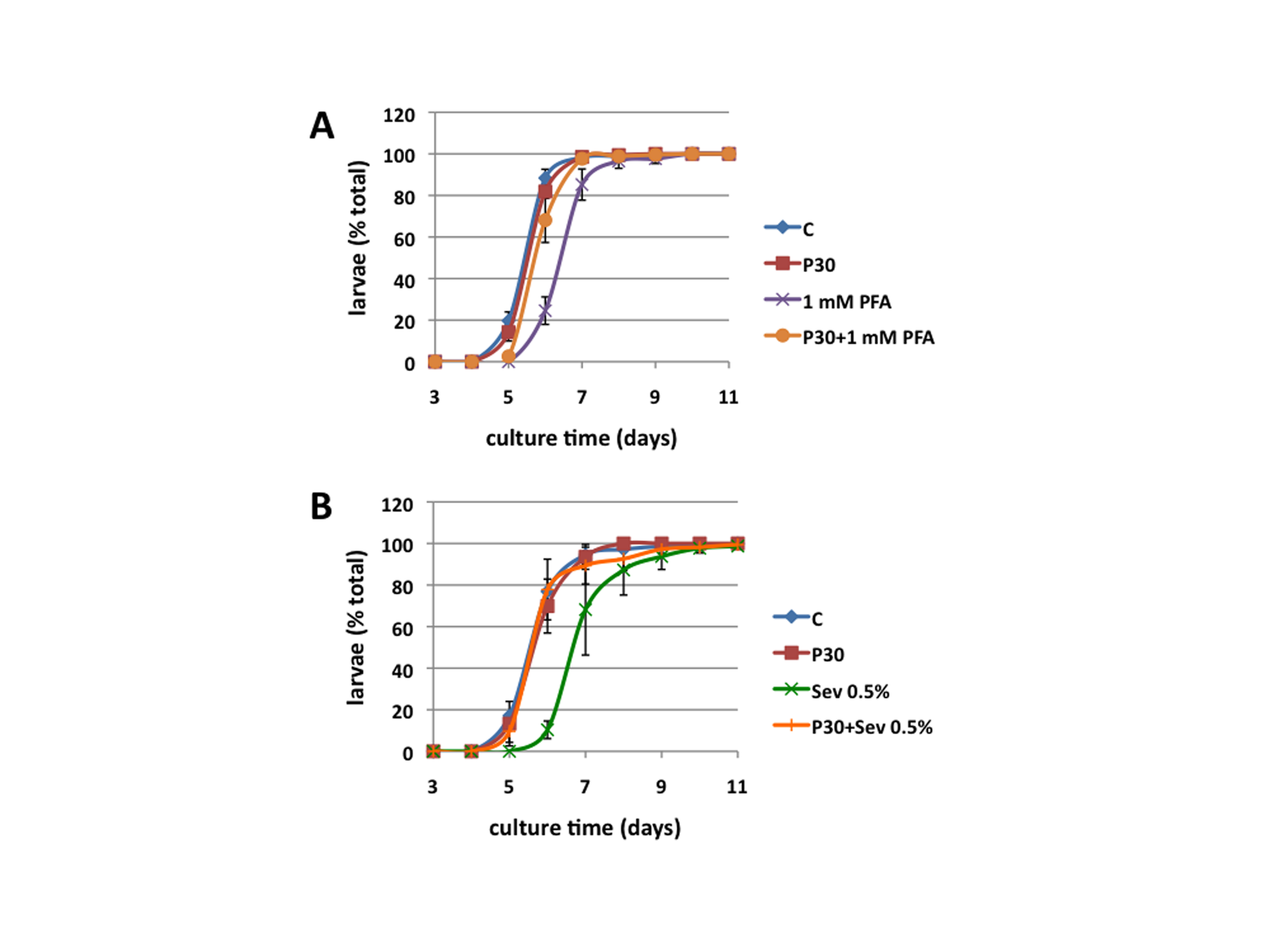

Supplement: Figure S4 — Phosphonoformic acid and sevelamer impair larval development. Yellow white flies were cultured on standard medium at 25°C. This medium was supplemented with 30 mM sodium-phosphate (pH6.0)(P30), 1 mM phosphonoformic acid (PFA), or 0.5% sevelamer (Sev) or in combinations thereof. Number of larvae emerged from the medium over time are shown. (TIF) [file pone.0031730.s004.tif]
