# Supplementary material for: Roles of Major Facilitator Superfamily Transporters in Phosphate Response in Drosophila
Source: PLoS One. 2012 Feb 16;7(2):e31730. doi: 10.1371/journal.pone.0031730 (PMC3280997; doi:10.1371/journal.pone.0031730)

A

		pho84 YML123C	*MFS16* FBpp0071516	*MFS18* FBpp0077146	*MFS17* FBpp0112571	*MFS3* FBpp0077623	*MFS13* FBpp0079876	*MFS10* FBpp0073555	*MFS15* FBpp0085818	*MFS14* FBpp0085817
pho84	YML123C	100.0% (587 AA)								
MFS16	FBpp0071516	11.3% (645 AA)	100.0% (566 AA)							
MFS18	FBpp0077146	10.9% (595 AA)	12.3% (583 AA)	100.0% (439 AA)						
MFS17	FBpp0112571	7.6% (604 AA)	12.2% (582 AA)	17.7% (479 AA)	100.0% (445 AA)					
MFS3	FBpp0077623	10.4% (616 AA)	13.0% (632 AA)	18.4% (528 AA)	22.4% (532 AA)	100.0% (512 AA)				
MFS13	FBpp0079876	11.9% (597 AA)	14.1% (618 AA)	17.7% (509 AA)	20.2% (516 AA)	27.5% (523 AA)	100.0% (496 AA)			
MGF10	FBpp0073555	12.9% (628 AA)	13.9% (617 AA)	19.4% (568 AA)	22.0% (563 AA)	26.5% (589 AA)	29.5% (584 AA)	100.0% (559 AA)		
MFS15	FBpp0085818	12.5% (592 AA)	14.3% (614 AA)	19.2% (516 AA)	21.7% (531 AA)	27.8% (543 AA)	29.5% (522 AA)	29.7% (582 AA)	100.0% (497 AA)	
MFS14	FBpp0085817	10.2% (598 AA)	13.0% (614 AA)	19.4% (509 AA)	21.8% (514 AA)	28.7% (523 AA)	29.1% (515 AA)	27.6% (572 AA)	45.2% (524 AA)	100.0% (497 AA)

B

		pho84 YML123C	*MFS16* FBpp0071516	*MFS18* FBpp0077146	*MFS17* FBpp0112571	*MFS3* FBpp0077623	*MFS13* FBpp0079876	*MFS10* FBpp0073555	*MFS15* FBpp0085818	*MFS14* FBpp0085817
pho84	YML123C	100.0% (587 AA)								
MFS16	FBpp0071516	27.0% (100 AA)	100.0% (566 AA)							
MFS18	FBpp0077146	28.6% (126 AA)	100.0% (439 AA)							
MFS17	FBpp0112571	19.8% (86 AA)	19.1% (147 AA)	23.1% (350 AA)	100.0% (445 AA)					
MFS3	FBpp0077623	18.9% (122 AA)	24.4% (168 AA)	24.9% (389 AA)	27.2% (444 AA)	100.0% (512 AA)				
MFS13	FBpp0079876	18.5% (157 AA)	24.4% (127 AA)	23.9% (397 AA)	27.5% (411 AA)	30.6% (487 AA)	100.0% (496 AA)			
MGF10	FBpp0073555	27.5% (109 AA)	21.9% (151 AA)	26.9% (417 AA)	28.5% (445 AA)	31.8% (510 AA)	37.6% (484 AA)	100.0% (559 AA)		
MFS15	FBpp0085818	21.1% (133 AA)	19.2% (468 AA)	23.7% (438 AA)	28.6% (413 AA)	32.4% (485 AA)	32.6% (460 AA)	36.5% (490 AA)	100.0% (497 AA)	
MFS14	FBpp0085817	22.1% (145 AA)	23.1% (208 AA)	24.8% (396 AA)	28.6% (413 AA)	30.6% (497 AA)	32.5% (427 AA)	33.3% (493 AA)	51.1% (464 AA)	100.0% (497 AA)

C

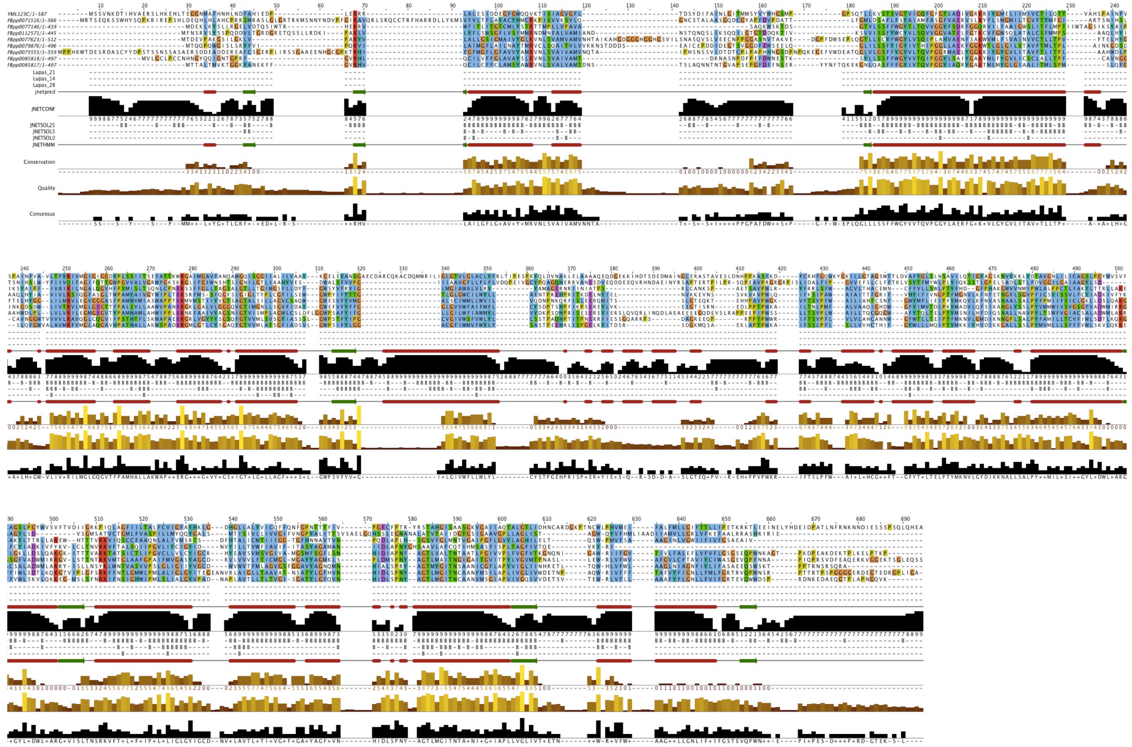

Supplement: Figure S2 — Sequence comparison between ph84 and MFS transporters expressed in S2R+ cells. A: Global alignment. Amino acid sequence identity in % between the sequence shown in column and row (alignment length in brackets). B: Local alignment. Amino acid sequence identity in % between sequence shown in column and row (alignment length in brackets). C: Clustal W alignment of fly transporters expressed in S2R+ cells along with Pho84 using Jalview (http://www.jalview.org/download.html). (PDF) [file pone.0031730.s002.pdf]
